# *De novo* assembly and characterization of transcriptomes of early-stage fruit from two genotypes of *Annona squamosa* L. with contrast in seed number

**DOI:** 10.1186/s12864-015-1248-3

**Published:** 2015-02-14

**Authors:** Yogesh Gupta, Ashish K Pathak, Kashmir Singh, Shrikant S Mantri, Sudhir P Singh, Rakesh Tuli

**Affiliations:** National Agri-Food Biotechnology Institute (NABI), Department of Biotechnology (DBT), C-127, Industrial Area, Phase-8, -160071 Mohali, India; University Institute of Engineering and Technology, Panjab University, Chandigarh, India

**Keywords:** *Annona squamosa* L. transcriptomics, Early-stage developing fruit, *De novo* transcriptome assembly, Simple sequence repeats, Web resource

## Abstract

**Background:**

*Annona squamosa* L., a popular fruit tree, is the most widely cultivated species of the genus *Annona*. The lack of transcriptomic and genomic information limits the scope of genome investigations in this important shrub. It bears aggregate fruits with numerous seeds. A few rare accessions with very few seeds have been reported for *Annona*. A massive pyrosequencing (Roche, 454 GS FLX+) of transcriptome from early stages of fruit development (0, 4, 8 and 12 days after pollination) was performed to produce expression datasets in two genotypes, Sitaphal and NMK-1, that show a contrast in the number of seeds set in fruits. The data reported here is the first source of genome-wide differential transcriptome sequence in two genotypes of *A. squamosa*, and identifies several candidate genes related to seed development.

**Results:**

Approximately 1.9 million high-quality clean reads were obtained in the cDNA library from the developing fruits of both the genotypes, with an average length of about 568 bp. Quality-reads were assembled *de novo* into 2074 to 11004 contigs in the developing fruit samples at different stages of development. The contig sequence data of all the four stages of each genotype were combined into larger units resulting into 14921 (Sitaphal) and 14178 (NMK-1) unigenes, with a mean size of more than 1 Kb. Assembled unigenes were functionally annotated by querying against the protein sequences of five different public databases (NCBI non redundant, *Prunus persica*, *Vitis vinifera*, *Fragaria vesca*, and *Amborella trichopoda*), with an E-value cut-off of 10^−5^. A total of 4588 (Sitaphal) and 2502 (NMK-1) unigenes did not match any known protein in the NR database. These sequences could be genes specific to *Annona* sp. or belong to untranslated regions. Several of the unigenes representing pathways related to primary and secondary metabolism, and seed and fruit development expressed at a higher level in Sitaphal, the densely seeded cultivar in comparison to the poorly seeded NMK-1. A total of 2629 (Sitaphal) and 3445 (NMK-1) Simple Sequence Repeat (SSR) motifs were identified respectively in the two genotypes. These could be potential candidates for transcript based microsatellite analysis in *A. squamosa*.

**Conclusion:**

The present work provides early-stage fruit specific transcriptome sequence resource for *A. squamosa*. This repository will serve as a useful resource for investigating the molecular mechanisms of fruit development, and improvement of fruit related traits in *A. squamosa* and related species.

**Electronic supplementary material:**

The online version of this article (doi:10.1186/s12864-015-1248-3) contains supplementary material, which is available to authorized users.

## Background

*Annona squamosa* L., commonly known as sugar-apple (or sweetsop or custard-apple), is a popular fruit throughout the tropics, mainly southern Mexico, Antilles, Central and South America, tropical Africa, Australia, India, Indonesia, Polynesia and US (Hawaii and Florida) [1]. It is native to the tropical America and West Indies. In India, it was introduced by the Spanish and Portuguese in the 16th century [1,2]. It is known by several names in India: ata, aarticum, shareefa, sitaphal, seethaphal or seetha pazham, aathachakka and atna kothal etc. [1]. *Annona* sp. belongs to family Annonaceae which is the largest living family among magnoliids (primitive angiosperms). The genus *Annona* contains about 166 species [3], out of which six produce edible fruits; *A. squamosa*, *A. reticulata*, *A. cherimola*, *A. muricata*, *A. atemoya* and *A. diversifolia* [4]. *A. squamosa* is the most widely cultivated species [5]. The flower of *A. squamosa* comprises of a gynoecium of several loosely cohering carpels, surrounded by an androecium of numerous stamens, encircled by three small, inconspicuous sepals, and three green colored fleshy petals [6]. It is an apocarpous flower i.e. carpels are separate in individual pistils. Fruit is a syncarpium i.e. formed by amalgation of many ripened pistils and a fleshy receptacle. Each carpel has a single anatropous ovule that may develop into a single seed. The pulp is creamy white to light yellow, sweetly aromatic, and tastes like custard. The pulp is of nutritional and medicinal value [7,8], rich in calories, vitamin C, and minerals [1,9,10]. *Annona* fruits have been mentioned as ‘one of the most delicious fruits known to man’ and as ‘aristocrat of fruits’, considering its nutritional and medicinal value [11,12].

There have been very few genomic studies on *A. squamosa*, as only 158 and 12 sequences are available in nucleotide and protein databases, respectively, in NCBI GenBank as on 20th December, 2014 (http://www.ncbi.nlm.nih.gov/Taxonomy/Browser/wwwtax.cgi?id=301693). Next generation sequencing (NGS) technologies have facilitated rapid investigation of transcriptome [13-16]. The GS FLX+ platform is a high-throughput system, which can generate long sequence reads (up to ~1 kb), with high accuracy (http://454.com/products/gs-flx-system). We report *de novo* assembly and transcriptome catalogue from *A. squamosa*. The data provides an important resource for gene discovery, gene expression, functional analysis, molecular breeding, and comparative genomic analysis of *A. squamosa* and related species.

In most angiosperms, including *A. squamosa*, ovule and ovary develop into seed and fruit, respectively. This transition is a complex physiological process with coordinated development of maternal and filial tissues. Understanding the early phase of fruit development is important, since the molecular and biochemical pathways of seed and fruit set, soon after fertilization, determine seed number, fruit size, and other fruit quality traits such as accumulation of sugar and organic acids [17-19]. Less number of seeds in fruit or seedlessness is important to consumers, both for fresh fruit consumption and fruit processing, especially when the seeds in *Annona* are hard and have a bad taste. Differences in fruit related traits, such as seed number have been reported among the *Annona* species and cultivars [9]. The presence of parthenocarpic fruits has not been reported in *Annona* sp. However, absence of the outer integument and change in ovule structure have been suggested as the causes for failure in seed formation due to interruption in the reproductive program in a spontaneous mutant of *A. squamosa* (*Thai seedless*) [11]. In India, some accessions have been reported with significantly reduced number of seeds, as compared to the common sugar-apple, Sitaphal [20,21]. In order to gain molecular insight into early-stage fruit development and to create groundwork for molecular characterization of fruit development, it is desirable to profile the transcriptome of developing fruits of *A. squamosa*.

In the present study, a massive pyrosequencing of transcriptome from early stages of fruit development was performed in two *Annona* genotypes (Sitaphal and NMK-1), showing significant difference in fruit seed number, using NGS technology (Roche 454 GS FLX+). *De novo* transcriptome assembly, functional annotation, and *in silico* discovery of potential molecular markers have been described here. Various genes, related to hormone, seed and fruit development, transcription factors, and metabolic pathways were identified. The information will be helpful in functional genomic studies and in furthering the understanding of molecular mechanisms of fruit development in *Annona* sp.

## Methods

### Plant material and RNA extraction

**Figure 1:**
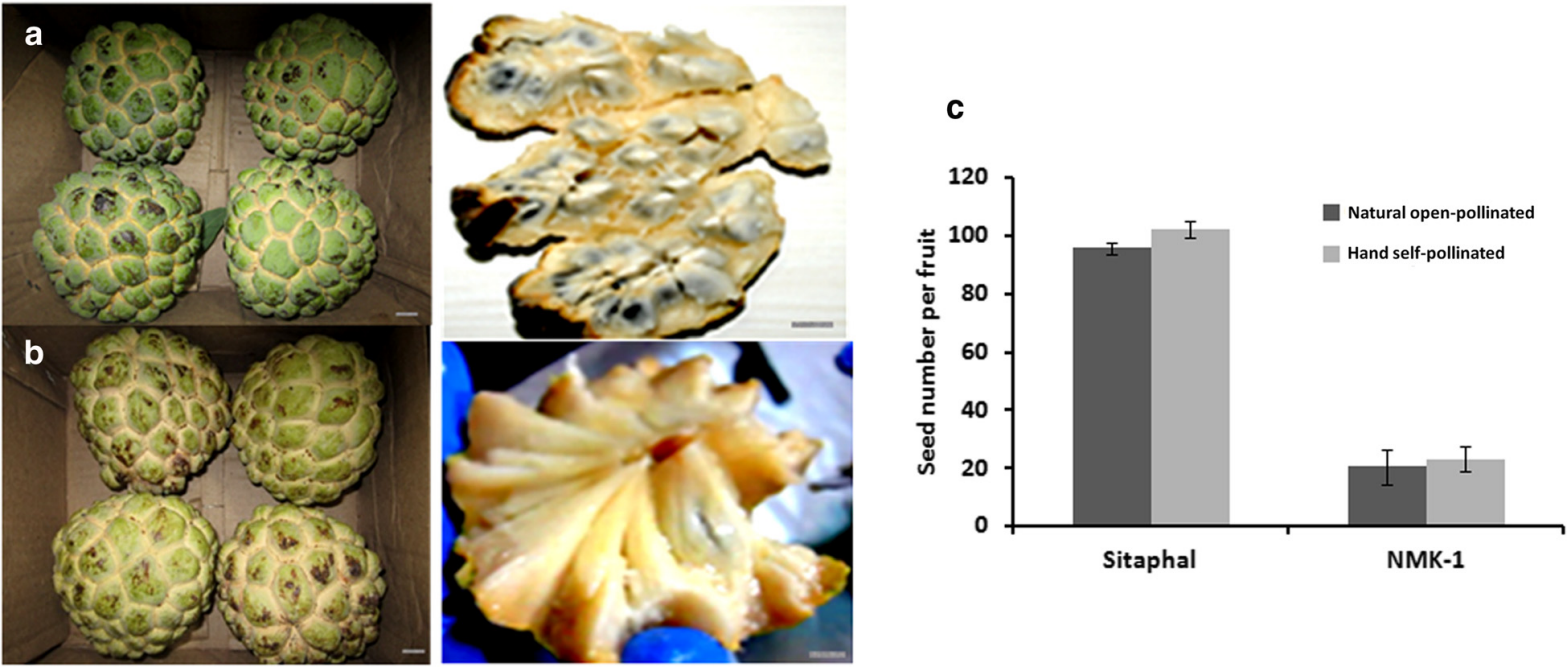
**Mature fruits of Sitaphal (a) and NMK-1 (b), showing densely seeded and nearly seedless ripened carpels (Scale 2 cm), respectively.** Bar diagram shows the difference between the two genotypes in fruit seed number **(c)**. The error bars indicate standard error in thirty mature fruits, harvested from three different plants (10 fruits from each plant) of each genotype.

Two *Annona* genotypes with contrast in fruit seed number (Figure 1), Sitaphal and NMK-1, were used in this study. Sitaphal is a well known cultivar of *A. squamosa* [22]. NMK-1 was developed by selection for desirable characteristics from a population of *Annona* genotypes [21]. However, systematic information on the development of the cultivars is not available. Phylogenetic analysis, using two marker sequences (*rbcl* and LMCH10) in seventeen species of *Annona*, placed both the genotypes close to *A. squamosa* (Additional file 1: Figure S1). The two genotypes were collected from the field of Madhuban Nursery (17.68° N 75.92° E coordinates, at an elevation of 457 m), Solapur, Maharshtra, India, where these are clonally propagated.

**Figure 2:**
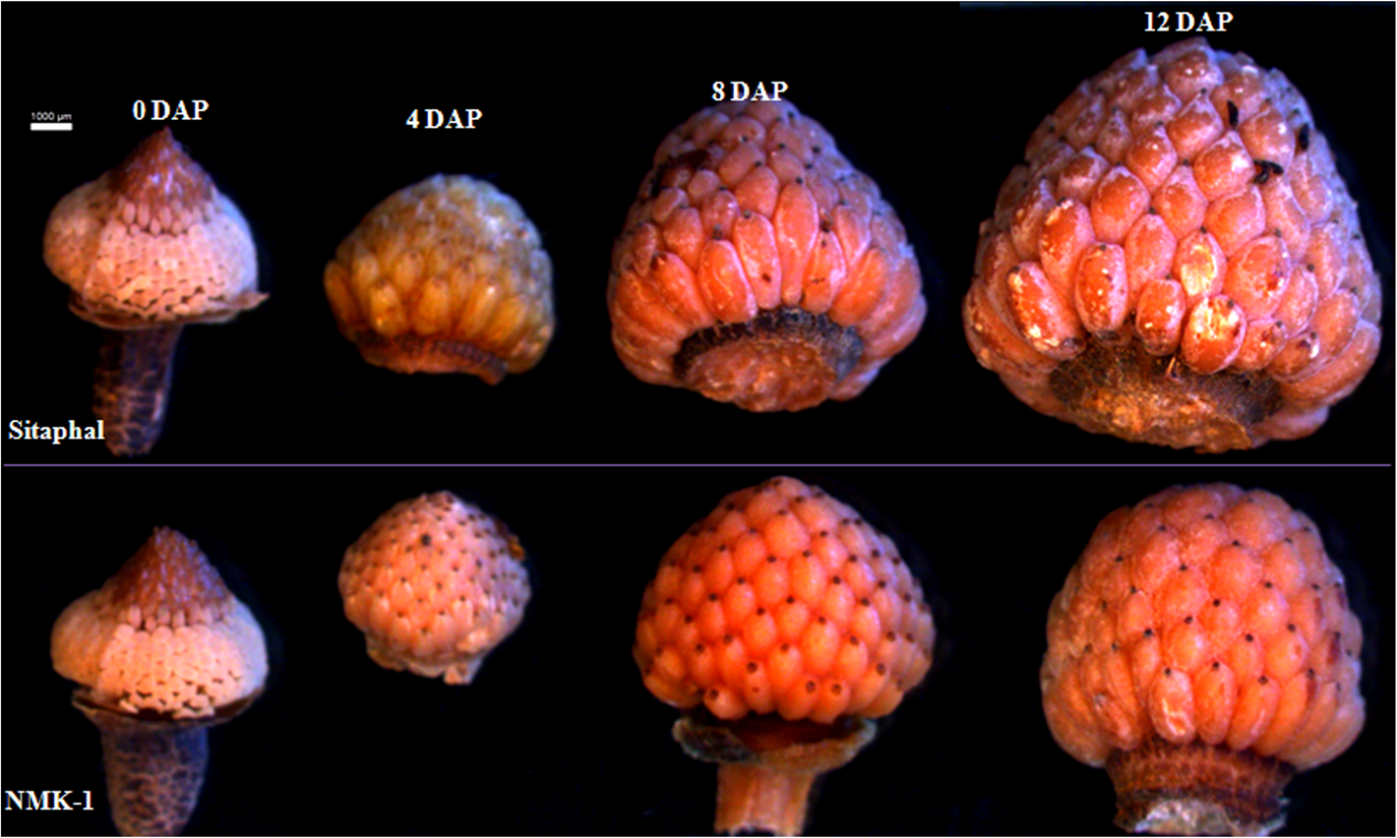
**Early-stage developing fruits (0, 4, 8, and 12 DAP) in Sitaphal and NMK-1.**

Pollens were collected from flowers, in male stage, as described by Jalikop and Kumar [4]. The flowers, in female stage, were hand self-pollinated, using freshly collected pollens, in the morning (06.00 and 10.00 h). All the flowers were pollinated at the same time to avoid confounding effect of environment on fruit development. In each pollinated flower, the floral tube was plugged with cotton to prevent contamination of outside pollen. Flowers in similar stages were tagged and left as un-pollinated controls to examine seed numbers in fruits, developed from hand self-pollination and natural open-pollination (Figure 1c). The experiment was performed on three plants (three biological replicates) each of both the genotypes, during July, 2012. Developing fruits were harvested at 4, 8, and 12 days after pollination (DAP) (Figure 2). The gynoecium comprising of unfertilized ovules (0 DAP) was harvested. All the stamens were removed surrounding the gynoecium before harvesting. The 0, 4, 8 and 12 DAP samples were surface sterilized by using absolute ethanol before harvesting. The samples were frozen in liquid nitrogen immediately after harvest, and stored at −80°C until use.

Total RNA was isolated from the developing fruits (hand self-pollinated) using RNA isolation kit (Sigma) following the manufacturer’s instructions. For RNA extraction, at least three developing fruits of same stage (0, 4, 8, and 12 DAP) were taken for each sample. The RNA was extracted in three biological replicates for each genotype. The quality of RNA was confirmed by using 2100 Bioanalyzer (Agilent). For sequencing, in case of each sample (0, 4, 8, and 12 DAP of Sitaphal or NMK-1), the equivalent quantity of total RNA of three biological replicates was pooled.

### Library preparation and 454 pyrosequencing

Approximately 1 μg total RNA was used for preparing mRNA sequencing library of each sample. Poly (A^+^) RNA was isolated from total RNA mixture by using NEBNext® Poly(A) mRNA Magnetic Isolation Module (New England Biolabs), following the manufacturer’s protocol. The purified Poly (A^+^) RNA was sheared and cDNA library was prepared by using NEBNext® mRNA Library Prep Reagent Set for 454™ (New England Biolabs) as per the instruction manual. The cDNA libraries were sequenced on a 454 Genome Sequencer FLX+ platform (Roche, USA).

### *De novo* assembly

The raw data obtained from 454 GS FLX+ was filtered with a quality cut-off value of 40. The adaptor and primer sequences were removed using NGS QC Tool kit and GS Run processor. The low-quality sequences and sequences with less than 40 bp were removed before contig assembly. *De novo* contig assembly of the reads was performed using GS *De Novo* Assembler v2.6 (Roche, USA). In the assembled transcript sequence data at the four developmental stages (0, 4, 8 and 12 DAP), repeat sequences were identified with at least 40 bp overlap and 95% overlap identity. The repeat sequences of the transcriptome data from four libraries (0, 4, 8 and 12 DAP) were combined into larger units- unigenes, by using CAP3 [23].

### Functional annotation

Accelerated large scale functional annotation of all contigs was done using WImpiBLAST tool [24] on high performance computing cluster. For functional annotation, the assembled transcript sequences were used as queries against the non-redundant (NR) protein database using NCBI BLASTx algorithm [25]. The BLAST E-value threshold was set at 10^−5^.

### Gene ontology analysis

The gene ontology (GO) annotation was derived for the unigenes, using Uniprot annotation [26]. The categorization and visualization of GO terms was done using WEGO web tool [27].

### Comparative analysis of *A. squamosa* unigenes

Comparative analysis was performed using the unigenes as queries against the protein databases of some fruit crops such as, *Prunus persica* (http://www.rosaceae.org/species/prunus_persica/genome_v1.0), *Vitis vinifera* (ftp://ftp.psb.ugent.be/pub/plaza/plaza_public_02_5/Fasta/proteome.vvi.tfa.gz) and *Fragaria vesca* (ftp://ftp.psb.ugent.be/pub/plaza/plaza_public_02_5/Fasta/proteome.fve.tfa.gz), and a primitive angiosperm, *Amborella trichopoda* (http://amborella.huck.psu.edu/).

### Detection of sequences associated with hormone related signalling pathways, transcription factors and seed development

The unigene sequences were used to blast (BLASTx) against the transcription factor (http://planttfdb.cbi.edu.cn), seed development (http://www.seedgenes.org/) and hormone related (http://molbio.mgh.harvard.edu/sheenweb/Ara_pathways.html) protein sequence database of *Arabidopsis thaliana*, at the criteria of E-value ≤ 10^−5^ and query coverage ≥ 50%.

### Single nucleotide polymorphism (SNP) analysis

Reads from the transcriptome libraries were mapped on unigenes of the respective genotype using program 'clc_ref_assemble_long' of CLC Assembly Cell version 3.2.2. Variants were detected using 'find_variations' program. SNP with read depth of more than five for each allele was only considered as heterozygous.

### Detection of simple sequence repeats (SSRs)

The unigene sequences were searched for SSRs using the perl script program MISA (MIcroSAtellite; http://pgrc.ipk-gatersleben.de/misa/). The repeats of mono- to hexa-nucleotide motifs with a minimum of five repetitions were considered as search criteria in MISA script [15].

### Web resource

A web resource, comprising of entire transcriptome contigs, has been developed using open source sequence server [28], and was hosted on Linux server.

### Identification of differentially expressed unigenes

A total 5689 unigenes common between the two genotypes with more than 80% query coverage, were used as reference sequences for mapping individual reads from each library. The calculation of reads per kilobase of transcript per million mapped reads (RPKM) was done by using the program 'clc_ref_assemble_long' of CLC Assembly Cell version 3.2.2.

Enrichment of Gene Ontology (GO) terms in the differentially expressed genes was performed using AgriGO analysis tool (http://bioinfo.cau.edu.cn/agriGO), with Fisher tests and Bonferroni multiple testing correction (False Discovery Rate ≤ 0.05). Kyoto Encyclopedia of Genes and Genomes (KEGG) categories was assigned by the plant gene set enrichment analysis toolkit (http://structuralbiology.cau.edu.cn/PlantGSEA/analysis.php) with fisher test function (False Discovery Rate ≤ 0.05).

### Quantitative real time PCR

First strand cDNA was synthesized using cDNA Synthesis Kit RT-PCR (Roche, USA), with oligodT anchored primers following the manufacturer’s instructions. Gene-specific primers were designed using Primer Express software. QuantiTect SYBR Green RT-PCR Master mix (Qiagen) was used to perform real time PCR assay in an ABI 7700 Sequence Detector Real-Time PCR system (Applied Biosystems, USA). Three biological replications were conducted for each transcript for both the genotypes. The expression data was analyzed using ABI PRISM 7700 Sequence Detection System software (Applied Biosystems). The expression values were normalized with respect to *Actin* gene from *A. squamosa*. Dissociation curves confirmed the presence of a single amplicon in each PCR reaction. Relative expression was calculated as described previously [29].

## Results and discussion

### 454 pyrosequencing, sequence assembly and annotation

In total, 1,801,608 and 1,901,179 raw reads were produced in the four cDNA library preparations of developing fruits (0, 4, 8 and 12 DAP) from the two genotypes of *A. squamosa*- Sitaphal and NMK-1 (Figure 2), respectively, with an average length of 568 bp (Additional file 2). The raw reads were filtered by removing low-quality reads, adapters, primer sequences, and sequences of less than 40 bp. Finally, 9,37,270 and 9,92,439 quality reads were obtained in the four cDNA library preparations (0, 4, 8 and 12 DAP) of Sitaphal and NMK-1, respectively. The average number of reads produced for each library was 0.24 million (Table 1). The filtered raw reads (sff files) were deposited in the NCBI Short Read Archive (SRA) database (accession number SRP042646). The quality-reads were assembled, giving 2074 to 11004 contigs, with more than 200 bp length, in the eight different cDNA libraries (Table 1). The contig sequences were searched against the known sequences in NCBI non redundant (NR) database, using BLASTx algorithm. At the E-value ≤ 10^−5^, 1808 to 9038 contigs were annotated across different libraries (Table 1, Additional file 3). The results provide sequence information for genes expressed during early developmental stages of fruits of *A. squamosa*.

**Table 1:** **Summary of the sequencing-reads, assembly and functional annotation (using NCBI NR database) of the** ***A*** **.** ***squamosa*** **transcriptome**

**Genotype**	**Developmental stage**	**Total high quality reads**	**Average read length**	**Total number of contigs (≥200 bp)**	**Total number of annotated contigs (≥200 bp)**
Sitaphal	0 DAP	227,732	635	10403	8176
	4 DAP	198,269	512	2074	1808
	8 DAP	219,057	574	6850	6023
	12 DAP	292,212	578	7394	6512
NMK1	0 DAP	288,216	592	8645	7401
	4 DAP	287,824	584	11004	9038
	8 DAP	272,750	589	7001	6003
	12 DAP	143,649	479	2078	1886

The details of the contigs are given in Additional file 3.

The contig sequence data in the four stages of fruit development (0, 4, 8, and 12 DAP) was combined into larger units, mentioned here as unigenes, by using CAP3. A total of 14921 (Sitaphal) and 14178 (NMK-1) unigenes were obtained. Out of the 14921 unigenes in Sitaphal, 2905 were ≥ 500 bp, 5239 were ≥ 1000 bp, 3663 were ≥ 1500 bp and 3114 were ≥ 2000 bp in length. Out of the 14178 unigenes in NMK-1, 2697 were ≥ 500 bp, 4883 were ≥ 1000 bp, 3516 were ≥ 1500 bp and 3082 were ≥ 2000 bp in length. The average lengths of the unigenes were 1086 bp and 1100 bp for Sitaphal and NMK-1, respectively. The sequence information is a useful resource for identification, cloning and functional genomic studies in future.

The 14178 unigenes of NMK-1 were mapped over 14921 unigenes of Sitaphal. A total of 5689 unigenes were common between the two genotypes with more than 80% query coverage. Single nucleotide polymorphism was investigated in the 1160 unigenes with at least 500 bp length and showing at least 95% similarity between the two genotypes. The SNP analysis estimated about 0.35 and 0.33% heterozygosity in Sitaphal and NMK-1, respectively, after examining about 2.2 and 1.3 million nucleotide positions. The low level of heterozygosity agrees with the previous reports, notifying the development of true-to-type and uniform seedlings in *A*. s*quamosa* [30,31].

### Functional categorization by GO annotation

**Figure 3:**
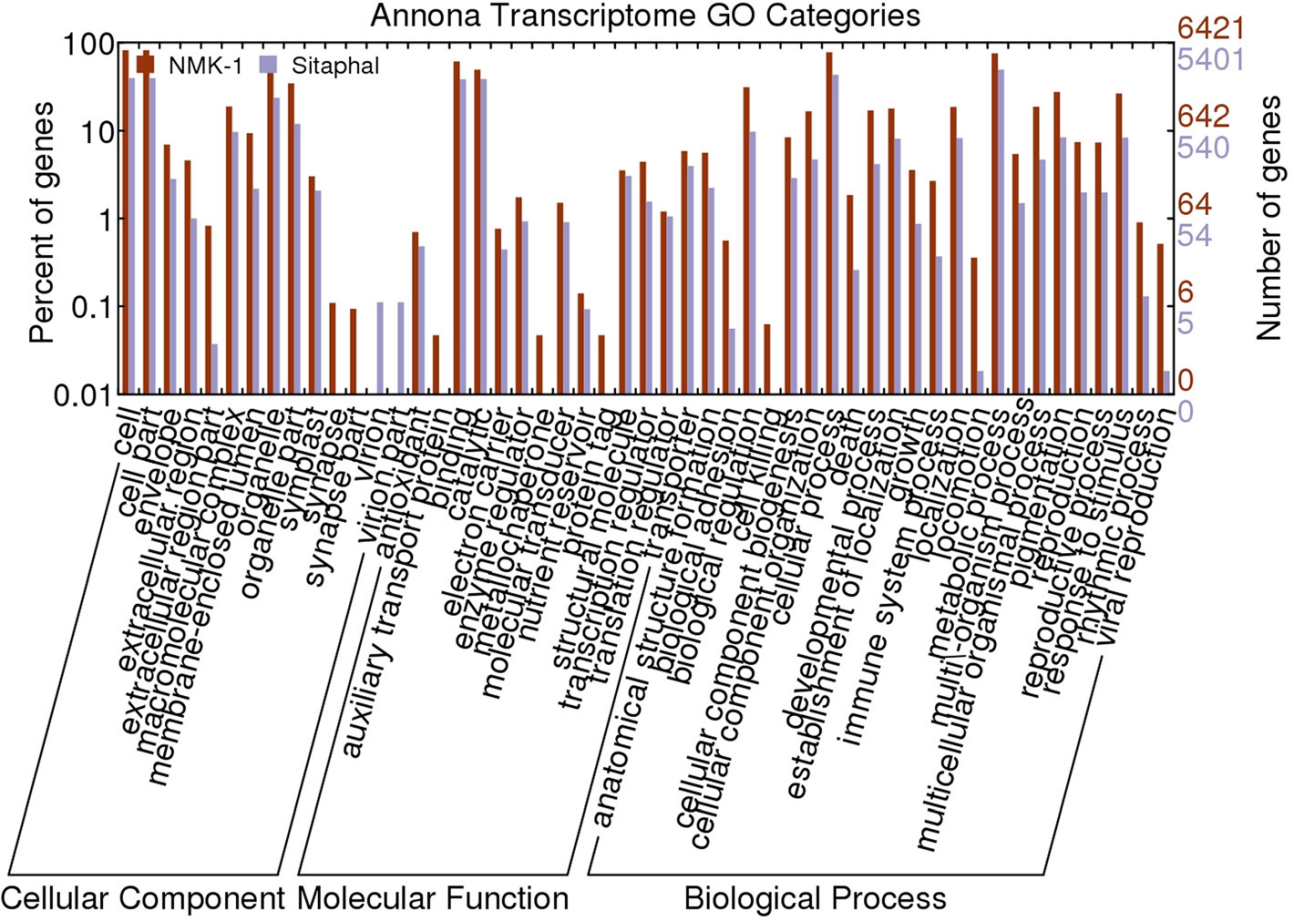
**GO classifications of assembled unigenes, having sequence homology with uniprot proteins, assigned to 51 functional groups.**

In total, 5401 (Sitaphal) and 6421 (NMK-1) unigenes, having sequence homology with uniprot annotations, were subjected to GO assignments for biological processes, cellular components and molecular functions categories. In the category of biological processes, unigenes related to metabolic processes (49.2% in Sitaphal and 75.3% in NMK-1), cellular processes (42.9% in Sitaphal and 77.3% in NMK-1), and response to stimulus (8.4% in Sitaphal and 26.2% in NMK-1) were predominant. In cellular components, genes related to cell parts (39.2% in Sitaphal and 81.8% in NMK-1) and organelles (23.5% in Sitaphal and 62.4% in NMK-1) were the most abundant classes. In molecular functions, genes involved in binding (38.1% in Sitaphal and 60.8% in NMK-1) and catalytic activities (38.3% Sitaphal and 49.1% in NMK-1) were abundantly expressed (Figure 3).

### Comparative analysis with available public databases

The assembled unigenes were functionally annotated using BLASTx algorithm against the protein sequences of five public databases: NCBI NR, *Prunus persica*, *Vitis vinifera* and *Fragaria vesca*, and a primitive species, *Amborella trichopoda*, with an E-value cut-off of 10^−5^. The number and percentage of annotated unigenes of *A. squmosa* genotypes are given in Table 2. Of the 14921 (Sitaphal) and 14178 (NMK-1) unigenes, 10333 (69.25%) and 11676 (82.35%), respectively, showed significant similarity to NCBI NR protein database (Additional file 4). Furthermore, 60.33% to 61.57% (Sitaphal) and 77.32% to 78.47% (NMK-1) of the unigenes showed significant homology with the four plant species (Table 2). We obtained 1928 (12.92%) and 2825 (19.92%) unigenes with more than 90% subject coverage, suggesting quasi-full length genes in Sitaphal and NMK-1, respectively. However, 4588 (Sitaphal) and 2502 (NMK-1) unigenes did not match with any known protein in the NR database. These un-assigned transcripts may be novel genes or belong to untranslated regions, and could play specific roles in *A. squamosa*. The unigene sequence information would be useful as a reference for molecular biology research on *A. squamosa* and its related species.

**Table 2:** **Number and percentage (in bracket) of unigenes in** ***A*** **.** ***squamosa*** **genotypes (Sitaphal and NMK-1) from BLASTx searches against public protein databases of fruits crop and a closely related genus**

**Database**	**Number of unigenes annotated in Sitaphal**	**Number of unigenes annotated in NMK-1**	**Number of unigenes with ≥90 subject coverage in Sitaphal**	**Number of unigenes with ≥90 subject coverage in NMK-1**
NCBI non redundant	10333 (69.25%)	11676 (82.35%)	1928 (12.92%)	2825 (19.92%)
*Vitis vinifera*	9187 (61.57%)	11126 (78.47%)	1557 (10.43%)	2289 (16.14%)
*Prunus persica*	9152 (61.33%)	11108 (78.34%)	1554 (10.41%)	2313 (16.31%)
*Fragaria vesca*	9218 (61.77%)	11206 (79.03%)	968 (6.48%)	1469 (10.36%)
*Amborella trichopoda*	9003 (60.33%)	10963 (77.32%)	1701 (11.40%)	2577 (18.17%)

### Detection of transcript sequences related to hormone pathway, transcription factors and seed development

Fruit development is a complex process and involves numerous physiological and biochemical events which are initiated and regulated by hormonal signals [32]. Plant hormones, such as auxins, gibberellins, cytokinins, abscisic acid, ethylene, and brassinosteroids, play important role in fruit set and development [17,33]. Brassinosteroids are important for early fruit development [34], and the regulation of seed size [35] and number [36]. A total of 148 unigenes encoding putative hormone related genes were identified in *A. squamosa* (Table 3, Additional file 5), by BLASTx searches against the protein database of hormone pathway genes of *A. thaliana*.

**Table 3:** **Summary of hormone related unigenes identified in the transcriptome of** ***A*** **.** ***squamosa*** **genotypes (Sitaphal and NMK-1)**

**Hormones**	**Number of unigenes**
Brassinosteroid	43
Auxin	31
Abscisic acid	28
Gibberellin	20
Ethylene	16
Cytokinin	10

The details of the unigenes are given in Additional file 5.

Transcription factors (TFs) control gene expression quantitatively, spatially and temporally [37]. It is desirable to identify the gene regulatory networks responsible for programming of early fruit development. The unigene sequences were annotated against the Plant-TFDB database of *A. thaliana*, to identify TFs which express during early phases of fruit development in *A. squamosa*. The BLASTx searches revealed a total of 319 unigenes matched with putative homologs of *Arabidopsis* TFs (Table 4, Additional file 6). The three most abundant families of transcription factors were bHLH, MYB and MYB-related, and C3H, represented by 34, 34 and 25 unigenes, respectively. Basic helix–loop–helix (bHLH) proteins participate in the regulation of a myriad of essential developmental and physiological processes, including reproductive development, determination of plant organ size, fruit and seed development [38,39]. The interplay of MYB factors, apart from transcription control on many crucial biological processes, regulates fruit and seed development [40,41]. Some of the C3H type TFs are embryo specific and play regulatory role in seed development [42].

**Table 4:** **Summary of transcription factor related unigenes identified in the transcriptome of** ***A*** **.** ***squamosa*** **genotypes (Sitaphal and NMK-1)**

**Transcription factor family**	**Numbers of unigenes**
bHLH	34
C3H	25
MYB	17
MYB-related	17
b ZIP	15
NAC	15
HD-ZIP	14
C2H2	15
HB-other	10
GRAS	10
ARF	11
WRKY	14
Trihelix	8
Mikc	8
FAR1	8
G2-like	8
ARR-B	6
ERF	6
TALE	6
SBP	6
CO-like	5
HSF	5

The table includes only those transcription factors which represent at least 5 unigenes in the transcriptome data. The details of the unigenes are given in Additional file 6.

The BLAST search on the transcriptome data, using *Arabidopsis* protein sequences obtained from SeedGenes Project (http://www.seedgenes.org/), identified 379 transcripts associated with the development of seeds (Additional file 7).

The sequence information on TFs, hormone and seed development related putative genes will be useful in examining the differential expression in the two genotypes of *A. squamosa*, with contrasting trait related to fruit and seed development.

### Differentially expressed unigenes

The transcript abundance profile was examined for the 5689 unigenes common between the two genotypes, in the developing fruits at 0 and 8 DAP. At these stages, comparable numbers of contigs were identified in the two genotypes (Table 1). A total of 5504 unigenes were differentially expressed between the two genotypes in at least one time point (0 or 8 DAP) (Additional file 8). Among these, 1792 and 721 unigenes were up- and down-regulated, respectively, by ≥ 2 fold in Sitaphal at 8 DAP. By using the information of BLASTx searches against the protein database of *A. thaliana*, the differentially expressed unigenes (≥2 fold, 8 DAP) were mapped to terms in AgriGO and KEGG databases [43,44]. The GO enrichment patterns showed a disproportionate representation of unigenes involved in the biological process of reproductive structure, embryo, seed and fruit development in the two genotypes (Table 5, Additional file 9). The ontology analysis based on KEGG revealed the abundance of transcripts related to hormones, alkaloids, terpanoids, steroids, phenylpropanoids, spliceosome and other metabolic pathways in Sitaphal (Table 6, Additional file 9). The results indicate a distinctly more active primary and secondary metabolism in the early-stage fruits of Sitaphal as compared to the less seeded NMK-1. Hence, development of multiple seeds in Sitaphal was accompanied by a higher rate of metabolism in developing fruits.

**Table 5:** **AgriGO categories (False discovery rate ≤ 0.05) for putative genes up-regulated (≥2 fold) in early-stage fruits (8 DAP) of Sitaphal and NMK-1**

**GO Term**	**Ontology**	**Description**	**Sitaphal**	**NMK-1**
GO:0003006	P	reproductive developmental process	76	0
GO:0005975	P	carbohydrate metabolic process	63	0
GO:0005996	P	monosaccharide metabolic process	23	0
GO:0006066	P	alcohol metabolic process	32	0
GO:0006082	P	organic acid metabolic process	70	0
GO:0006091	P	generation of precursor metabolites and energy	30	0
GO:0006457	P	protein folding	33	0
GO:0006461	P	protein complex assembly	19	0
GO:0006508	P	proteolysis	69	0
GO:0006511	P	ubiquitin-dependent protein catabolic process	36	0
GO:0006519	P	cellular amino acid and derivative metabolic process	58	0
GO:0006605	P	protein targeting	19	0
GO:0006810	P	transport	128	0
GO:0006886	P	intracellular protein transport	40	0
GO:0006950	P	response to stress	147	77
GO:0006996	P	organelle organization	64	0
GO:0007017	P	microtubule-based process	17	0
GO:0007275	P	multicellular organismal development	138	0
GO:0008104	P	protein localization	52	0
GO:0008152	P	metabolic process	591	230
GO:0008610	P	lipid biosynthetic process	40	0
GO:0009266	P	response to temperature stimulus	41	23
GO:0009408	P	response to heat	0	11
GO:0009628	P	response to abiotic stimulus	123	53
GO:0009790	P	embryonic development	45	0
GO:0009791	P	post-embryonic development	87	28
GO:0009987	P	cellular process	696	251
GO:0010035	P	response to inorganic substance	28	0
GO:0010154	P	fruit development	45	0
GO:0010876	P	lipid localization	0	7
GO:0015031	P	protein transport	49	0
GO:0016043	P	cellular component organization	97	0
GO:0016192	P	vesicle-mediated transport	29	0
GO:0019318	P	hexose metabolic process	18	0
GO:0019538	P	protein metabolic process	241	0
GO:0019752	P	carboxylic acid metabolic process	70	0
GO:0019941	P	modification-dependent protein catabolic process	36	0
GO:0022414	P	reproductive process	78	0
GO:0022607	P	cellular component assembly	32	0
GO:0032501	P	multicellular organismal process	144	0
GO:0032502	P	developmental process	156	0
GO:0033036	P	macromolecule localization	61	22
GO:0033365	P	protein localization in organelle	14	0
GO:0034613	P	cellular protein localization	42	0
GO:0034621	P	cellular macromolecular complex subunit organization	24	0
GO:0034637	P	cellular carbohydrate biosynthetic process	21	0
GO:0034641	P	cellular nitrogen compound metabolic process	52	0
GO:0042180	P	cellular ketone metabolic process	71	0
GO:0043170	P	macromolecule metabolic process	377	0
GO:0043436	P	oxoacid metabolic process	70	0
GO:0043632	P	modification-dependent macromolecule catabolic process	36	0
GO:0043933	P	macromolecular complex subunit organization	30	14
GO:0044085	P	cellular component biogenesis	54	0
GO:0044106	P	cellular amine metabolic process	41	0
GO:0044237	P	cellular metabolic process	506	197
GO:0044238	P	primary metabolic process	507	0
GO:0044248	P	cellular catabolic process	66	0
GO:0044257	P	cellular protein catabolic process	36	0
GO:0044260	P	cellular macromolecule metabolic process	337	0
GO:0044262	P	cellular carbohydrate metabolic process	43	0
GO:0044265	P	cellular macromolecule catabolic process	50	0
GO:0044267	P	cellular protein metabolic process	206	0
GO:0045184	P	establishment of protein localization	49	0
GO:0046907	P	intracellular transport	54	0
GO:0048316	P	seed development	44	0
GO:0048513	P	organ development	61	0
GO:0048608	P	reproductive structure development	75	0
GO:0048731	P	system development	61	0
GO:0048856	P	anatomical structure development	115	0
GO:0050896	P	response to stimulus	257	114
GO:0051179	P	localization	131	0
GO:0051234	P	establishment of localization	128	0
GO:0051603	P	proteolysis involved in cellular protein catabolic process	36	0
GO:0051641	P	cellular localization	62	0
GO:0051649	P	establishment of localization in cell	56	0
GO:0051716	P	cellular response to stimulus	62	0
GO:0065003	P	macromolecular complex assembly	28	0
GO:0070271	P	protein complex biogenesis	19	0
GO:0070727	P	cellular macromolecule localization	43	0

The details of the unigenes are given in Additional file 9.

**Table 6:** **Pathway assignment based on KEGG (False Discovery Rate ≤ 0.05) for putative genes up-regulated (≥2 fold) in early-stage fruits (8 DAP) of Sitaphal and NMK-1**

**Description**	**Sitaphal**	**NMK-1**
Metabolic pathways	152	63
Biosynthesis of plant hormones	47	0
Spliceosome	23	12
Biosynthesis of alkaloids derived from terpenoid and polyketide	28	0
Biosynthesis of terpenoids and steroids	31	0
Biosynthesis of alkaloids derived from shikimate pathway	28	0
Biosynthesis of alkaloids derived from ornithine, lysine and nicotinic acid	27	0
Proteasome	15	0
Citrate cycle (TCA cycle)	14	0
Biosynthesis of alkaloids derived from histidine and purine	24	0
Biosynthesis of phenylpropanoids	31	0
Inositol phosphate metabolism	10	0
Amino sugar and nucleotide sugar metabolism	14	0
Endocytosis	12	0
Aminoacyl-tRNA biosynthesis	10	0
Ribosome	19	0
Oxidative phosphorylation	13	0

The details of the unigenes are given in Additional file 9.

**Figure 4:**
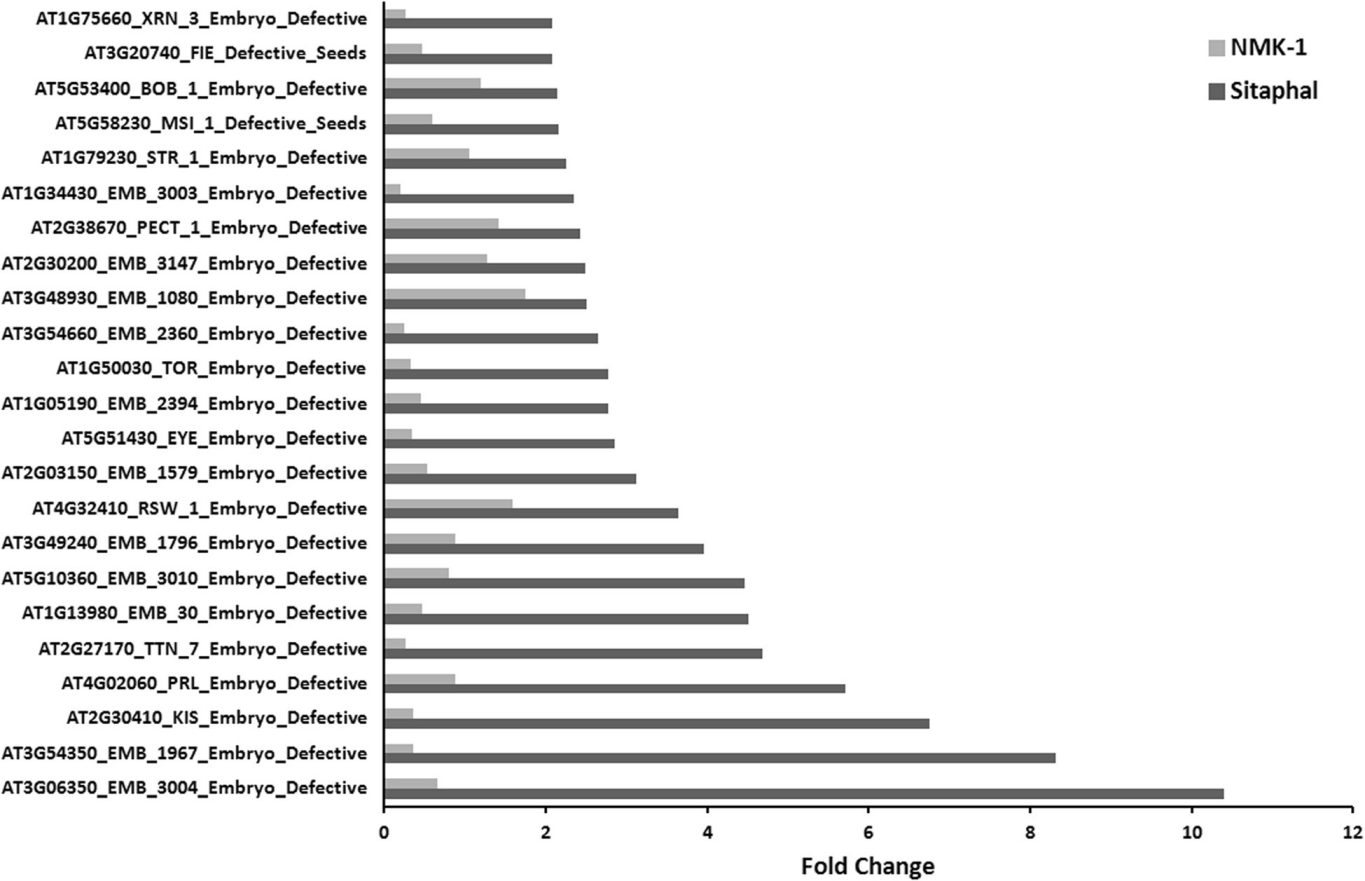
**Differential accumulation (≥2 fold, 8 DAP** ***vs*** **0 DAP) of transcripts for embryogenesis related putative genes in early-stage fruits of Sitaphal and NMK-1.** The orthologous genes give a defective embryo and/or seed phenotype in *Arabidopsis* mutants. The details of the differentially expressed transcripts are given in Additional file 8.

The transcript level of several unigenes associated with hormones, transcription factors, and seed development were also differentially expressed between the two genotypes at 0 and 8 DAP (Additional file 8). Many of the putative orthologous genes which give a defective embryo and/or seed phenotype in *Arabidopsis* mutants [45], showed reduced expression in NMK-1 at 8 DAP (Figure 4, Additional file 8). Lower level of expression of the embryogenesis-related genes (Figure 4) could be indicative of aberrant embryo development, eventually affecting seed development in NMK-1 plants. The underlying transcriptional changes need to be validated with the accompanying anatomical and metabolic changes in the developing ovules. Moreover, further in-depth RNA-sequencing is required to generate comprehensive transcriptional profile for each developing stage of fruits.

### Real time PCR

**Figure 5:**
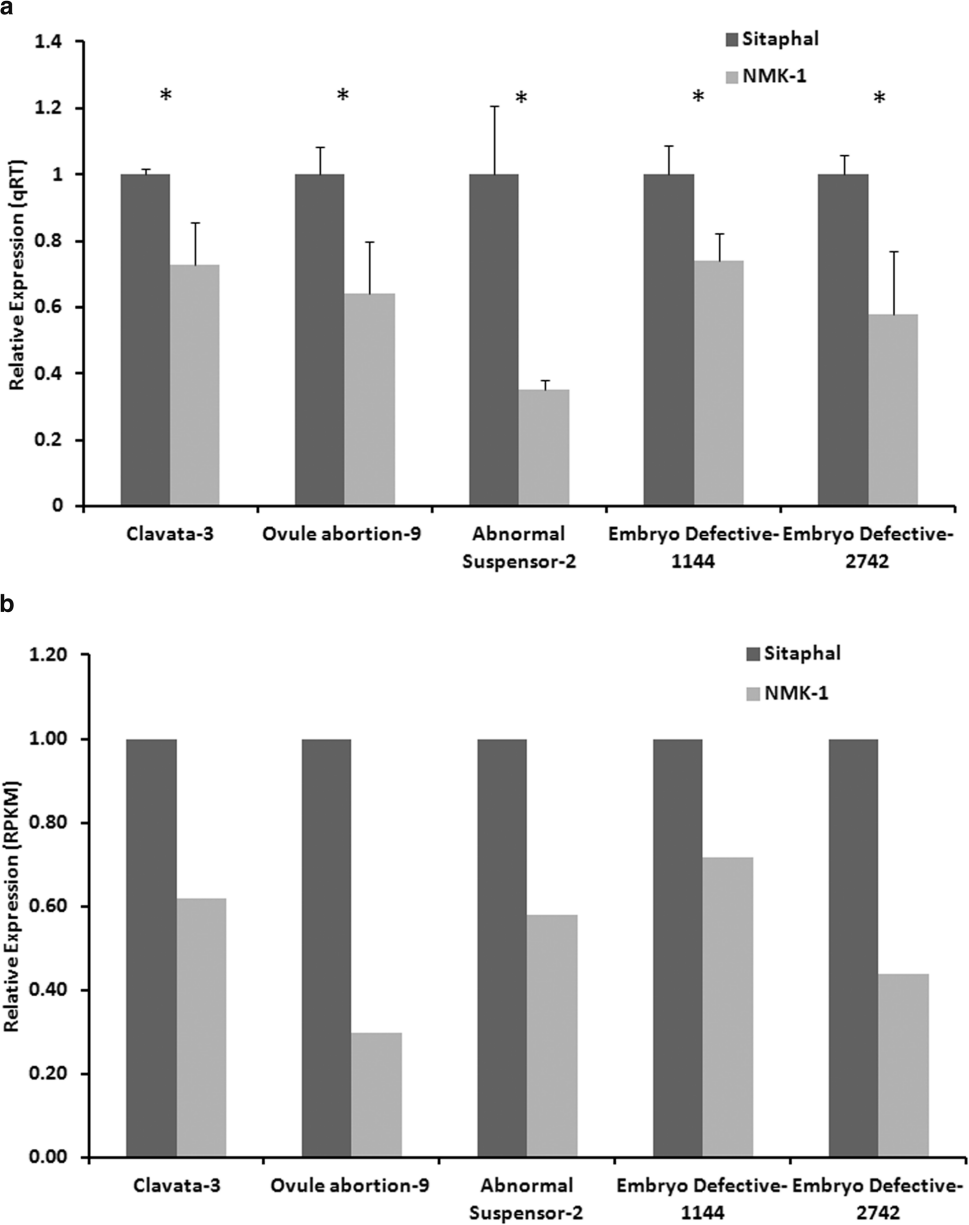
**Quantitative RT-PCR analyses and RPKM expression value of 5 randomly selected candidate genes for seed development in Sitaphal and NMK-1, at 8 DAP.** Quantitative RT-PCR analyses **(a)**. Each bar indicates standard error in three biological replicates (*p ≤ 0.05). A detail of the primers is given in Additional file 10. The qRT-PCR fold change is comparable with RPKM values in transcriptome data **(b)**.

To validate the usefulness of the transcript sequences identified in the transcriptome resource, expression of five randomly selected unigenes was examined by real time PCR and compared with the RPKM expression values in the transcriptome data of 5689 unigenes. qRT-PCR was performed in the developing fruit (8 DAP) of Sitaphal and NMK-1, in three biological replicates. At 8 DAP initial cell division takes place in the zygote, which leads to the formation of the embryo [46]. Interestingly, the qRT-PCR analysis (Figure 5a; Additional file 10) suggested preferentially lower expression of the orthologous genes such as *Clavata*-*3* (regulates seed formation [47]), *Abnormal Suspensor*-*2* (involved in embryogenesis [48]), *Embryo Defective*-*1144* (role in embryo development [49]), *Embryo Defective*-*2742* (role in embryo development [50]), and *Ovule abortion*-*9* (role in ovule development [51]). The qRT-PCR fold change was comparable to the RPKM values in the transcriptome data (Figure 5b). Thus, transcriptome data for the two contrasting *Annona* genotypes presented here is useful for identifying candidate genes for the development of less seeded fruits.

### Mining of SSRs

Identification of SSRs was carried out to generate information for the development of molecular markers for future studies on genetic diversity in *A. squamosa*. In total, 2629 and 3445 SSR motifs were identified in 2045 and 2678 transcripts for Sitaphal and NMK-1, respectively (Table 7). Out of the transcripts analyzed, 417 and 541 contained more than one SSR, whereas, 324 and 428 were in compound form in Sitaphal and NMK-1, respectively. The mono-nucleotide SSRs represented the largest fraction of SSRs (35.71% in Sitaphal and 44.06% in NMK-1), followed by di-nucleotide (30.88% in Sitaphal and 25.54% in NMK-1) and/or tri-nucleotide (29.25% in Sitaphal and 26.47% in NMK-1) SSRs. Tetra-, penta- and hexa-nucleotide SSRs were identified in a small fraction (0.030-0.004%) (Table 8). The 5689 unigenes, common between the two genotypes, were examined for the presence of SSRs with differences in length. In total, 18 SSRs were identified with variable number of tandem repeat loci between the two genotypes (Additional file 11). The SSR motifs could be potential candidates for transcript based microsatellite marker development, useful in determining functional genetic variation in *A. squamosa* [52].

**Table 7:** **Statistics of SSRs identified in the transcripts of** ***A*** **.** ***squamosa*** **genotypes (Sitaphal and NMK-1)**

**Statistics of SSRs**	**Sitaphal**	**NMK-1**
Total number of sequences examined	14921	14178
Total size of examined sequences	13101288	15606312
Total number of identified SSRs	2629	3445
Number of SSR containing sequences	2045	2678
Number of sequences containing more than one SSR	417	541
Number of SSRs present in compound Formation	324	428
Frequency of SSRs	4.53 kb / SSR	4.98 kb / SSR

**Table 8:** **Classes of SSR repeat motifs in the transcriptome of** ***A*** **.** ***squamosa*** **genotypes (Sitaphal and NMK-1)**

**Motifs**	**Sitaphal**	**NMK-1**
Mono-nucleotides	939 (35.71%)	1518 (44.06%)
Di-nucleotides	812 (30.88%)	880 (25.54%)
Tri-nucleotides	769 (29.25%)	912 (26.47%)
Tetra-nucleotides	81 (0.030%)	87 (0.025%)
Penta-nucleotides	12 (0.004%)	18 (0.005%)
Hexa-nucleotides	16 (0.006%)	30 (0.008%)

### *Annona* transcriptome web resource

A web resource has been developed where entire assembled transcripts are available in BLAST enabled search format (www.annonatranscriptome.nabi.res.in). The web resource is useful for researchers in data-mining and to access pre-computed annotations.

## Conclusion

The study provides transcriptome information on *A. squamosa*. We report sequencing, *de novo* assembly and analysis of early-stage fruit transcriptome of two genotypes with contrasting level of seed number in fruits. Orthologous genes related to hormone pathways, transcription factors and seed development were determined in the early-stage fruit tramscriptome. Differentially expressed unigenes were identified between the two genotypes. Several of such unigenes were related to seed and fruit related traits, and expressed at a higher level in the densely seeded genotype, Sitaphal. Additionally, a large number of SSRs were identified, which will be a useful resource in marker development for future genetic studies in *Annona* sp. This repository will serve as a useful resource for investigating the molecular mechanisms of fruit development, and improvement of fruit related traits in *A. squamosa* and related species.

### Availability of supporting data

The RNA-seq data is available in the NCBI Sequence Read Archive (SRA) (http://www.ncbi.nlm.nih.gov/sra), under accession number SRP042646.

## Additional files

Additional file 1: Figure S1. A phylogenetic tree on the basis of two markers- the chloroplast gene- *rbcl*, and the microsatellite locus- LMCH10. Additional file 2:
**Summary of 454 sequencing reads and contig assembly in developing fruit libraries of** ***A. squamosa*** **genotypes (Sitaphal and NMK-1).**
Additional file 3:
**Details of the contigs identified in the developing fruits (0, 4, 8 and 12 DAP) of** ***A. squamosa*** **genotypes (Sitaphal and NMK-1).**
Additional file 4:
**Details of the unigenes identified in the developing fruit transcriptome of** ***A. squamosa*** **genotypes (Sitaphal and NMK-1).**
Additional file 5:
**Details of hormone related unigenes identified in the developing fruit transcriptome of** ***A. squamosa*** **genotypes (Sitaphal and NMK-1).**
Additional file 6:
**Details of transcription factor related unigenes identified in the developing fruit transcriptome of** ***A. squamosa*** **genotypes (Sitaphal and NMK-1).**
Additional file 7:
**Details of seed development related unigenes identified in the developing fruit transcriptome of** ***A. squamosa*** **genotypes (Sitaphal and NMK-1).**
Additional file 8:
**Unigenes with RPKM values in the transcriptome data of developing fruits (0 and 8 DAP) in** ***A. squamosa*** **genotypes (Sitaphal and NMK-1).**
Additional file 9:
**AgriGO and KEGG categories (False Discovery Rate ≤ 0.05) for putative genes up-regulated (≥2 fold) in early-stage fruits (8 DAP) of Sitaphal and NMK-1.**
Additional file 10:
**Primers used in real time PCR analysis.**
Additional file 11:
**Polymorphic SSRs with variable number of tandem repeat loci in Sitaphal and NMK-1.**
