# Supplementary material for: De novo assembly and characterization of transcriptomes of early-stage fruit from two genotypes of Annona squamosa L. with contrast in seed number
Source: BMC Genomics. 2015 Feb 14;16(1):86. doi: 10.1186/s12864-015-1248-3 (PMC4336476; doi:10.1186/s12864-015-1248-3)
Supplement: Additional file 1: Figure S1. — A phylogenetic tree on the basis of two markers- the chloroplast gene- rbcl, and the microsatellite locus- LMCH10. [file 12864_2015_1248_MOESM1_ESM.pdf]

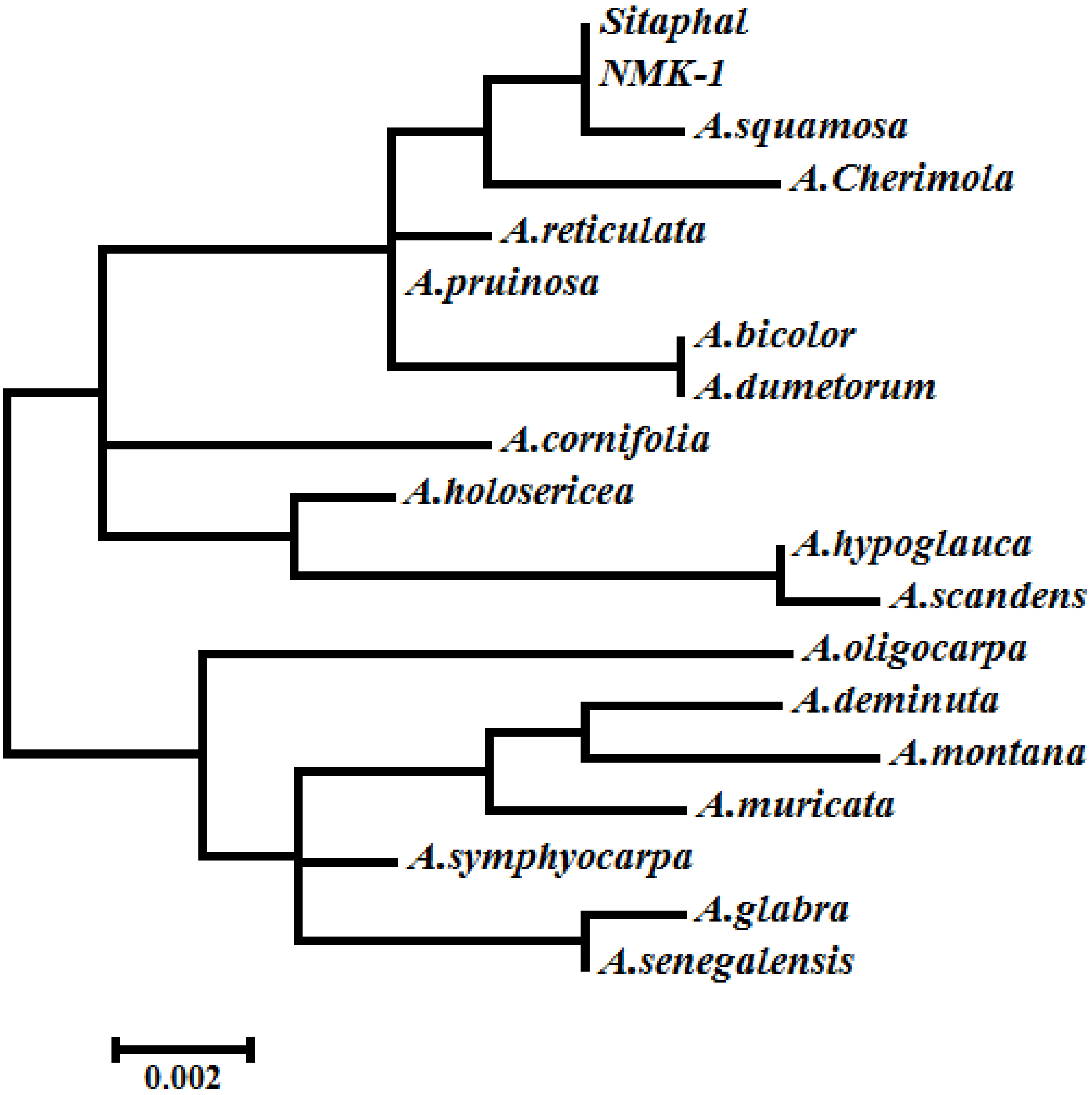

Figure S1. A phylogenetic tree on the basis of two markers- the chloroplast gene- *rbcl*, and the microsatellite locus- LMCH10. These have been demonstrated as promising markers for resolving phylogenetic relationship among the species of *Annona* [1]. The sequence information of the markers (*rbcl* and LMCH10) was extracted from NCBI for 16 species of *Annona*. The marker fragments were cloned and sequenced from *A. cherimola*, and the two cultivars- NMK-1 and Sitaphal. A combined data of the two marker sequences (*rbcl* and LMCH10) was used to construct a phylogenetic tree by Maximum Likelihood method based on the Tamura-Nei model in MEGA6 [2].

References:

1) Chatrou LW, Escribano MP, Viruel MA, Maas JW, Richardson JE, Hormaza JI: **Flanking regions of monomorphic microsatellite loci provide a new source of data for plant species-level phylogenetics**. *Mol Phylogenet Evol* 2009, **53**(3):726-733.

2) Tamura K, Stecher G, Peterson D, Filipski A, Kumar S: **MEGA6: Molecular Evolutionary Genetics Analysis version 6.0**. *Mol Biol Evol* 2013, **30**(12):2725-2729.
